# Imaging pathological tau in atypical parkinsonisms: A review

**DOI:** 10.1016/j.prdoa.2022.100155

**Published:** 2022-07-16

**Authors:** Anastassia M. Mena, Antonio P. Strafella

**Affiliations:** a Brain Health Imaging Centre, Campbell Family Mental Health Research Institute, Centre for Addiction and Mental Health, University of Toronto, Ontario, Canada; b Division of Brain, Imaging and Behaviour – Systems Neuroscience, Krembil Brain Institute, UHN, University of Toronto, Ontario, Canada; c Institute of Medical Science, University of Toronto, Ontario, Canada; d Edmond J. Safra Parkinson Disease Program & Morton and Gloria Shulman Movement Disorder Unit, Neurology Division, Dept. of Medicine, Toronto Western Hospital, UHN, University of Toronto, Ontario, Canada

**Keywords:** Parkinsonism, PSP, CBD, Parkinson’s disease, Neuroimaging, PET, SPECT, PSP, progressive supranuclear palsy, CBD, corticobasal degeneration, PET, positron emission tomography, SPECT, single-photon emission computerized tomography

## Abstract

Atypical parkinsonisms (APs) are a group of diseases linked to tau pathology. These include progressive supranuclear palsy (PSP) and corticobasal degeneration (CBD). In the initial stages, these APs may have similar clinical manifestations to Parkinson’s disease (PD) and other parkinsonisms: bradykinesia, postural instability, tremor, and cognitive decline. Because of this, one major hurdle is the accurate early diagnosis of APs. Recent advances in positron emission tomography (PET) radiotracer development have allowed for targeting pathological tau in Alzheimer’s disease (AD). Currently, work is still in progress for identifying a first-in-class radiotracer for imaging tau in APs. In this review, we evaluate the literature on in vitro and in vivo testing of current tau PET radiotracers in APs. The tau PET tracers assessed include both first-generation tracers ([18F]AV-1451, [18F]FDDNP, [18F]THK derivatives, and [11C]PBB3) and second-generation tracers ([18F]PM-PBB3, [18F]PI-2620, [18F]RO-948, [18F]JNJ-067, [18F]MK-6240, and [18F]CBD-2115). Concerns regarding off-target binding to cerebral white matter and the basal ganglia are still prominent with first-generation tracers, but this seems to have been mediated in a handful of second-generation tracers, including [18F]PI-2620 and [18F]PM-PBB3. Additionally, these two tracers and [18F]MK-6240 show promising results for imaging PSP- and CBD-tau. Overall, [18F]AV-1451 is the most widely studied tracer but the mixed results regarding its efficacy for use in imaging AP-tau is a cause for concern moving forward. Instead, future work may benefit from focusing on the second-generation radiotracers which seem to have a higher specificity for AP-tau than those originally developed for imaging AD-tau.

## Highlights

• [18F]AV-1451 displays mixed results for specificity to 4R CBD- and PSP-tau.
• [18F]PI-2620 and [18F]PM-PBB3 are the most promising second-generation tau PET tracers.
• Research using second-generation tau PET tracers in CBD and PSP is still limited.
• Finding an imaging diagnostic biomarker requires further work with larger samples.

## 1 Introduction

Parkinsonism is classified as the combination of bradykinesia with symptoms such as tremor, rigidity, and postural instability [1]. The most prevalent parkinsonism worldwide is Parkinson’s disease (PD). PD is a neurodegenerative disorder that is characterized by movement impairment, cognitive decline, behavioral complications, and decreased somatosensory function [2]. It is a common disease that affects > 2 % of aged individuals over 85 in North America [1]. PD symptomology arises due to misfolded alpha-synuclein build up and dopamine depletion in the substantia nigra which hinders voluntary movement [1]. A group of diseases with sometimes similar parkinsonian features fall under the category of atypical parkinsonisms (APs) [3], [4]. These include progressive supranuclear palsy (PSP), corticobasal degeneration/syndrome (CBD/S), dementia with Lewy bodies (DLB), and multiple system atrophy (MSA) [3].

PSP and CBD are considered tauopathies: neurodegenerative diseases in which the tau protein is misfolded and forms aggregates. PSP patients present with hindered postural stability, causing affected individuals to have frequent falls, and vertical gaze palsy [5], [6]. It is typically considered a sporadic neurodegenerative disease but increasing observations seem to also suggest a genetic association [7]. PSP is the second most common parkinsonism, following PD, with estimated prevalence ranging from 5 to 17/100,000 patients in their mid-60 s [8], [9]. This large range in prevalence is due to the heterogeneity of disease phenotypes and diagnostic criteria.

CBD is a sporadic atypical parkinsonism that presents itself in several different ways, most notably as corticobasal syndrome (CBS). CBD is characterized by various motor disorders such as akinesia, rigidity, and dystonia that predominate on one side of the body, accompanied by cortical defects (e.g., sensory dysfunction, apraxia, and alien-limb phenomenon) [6]. The heterogeneity of disease presentation contributes to clinicians’ difficulty with accurate diagnoses [10]. At present, the most reliable diagnostic method for CBD is through pathological confirmation upon post-mortem examination.

During the initial disease stages, patients with PSP and CBD may present with similar clinical features to PD, but they have additional symptoms that are not easily controlled by levodopa [11]. Therefore, there is a dire need for the development of biomarkers capable of differential diagnosis. The production of such diagnostic biomarkers will help speed up the diagnostic process and the development of disease-modifying treatments for these diseases. Over the last decade the creation and assessment of various tau positron emission tomography (PET) radiotracers has been a promising avenue for identifying diagnostic biomarkers [12]. In this review, we summarize the key findings of studies examining tau PET tracers and their efficacy for imaging tau in APs.

## 2 Pathological tau

**Fig. 1:**
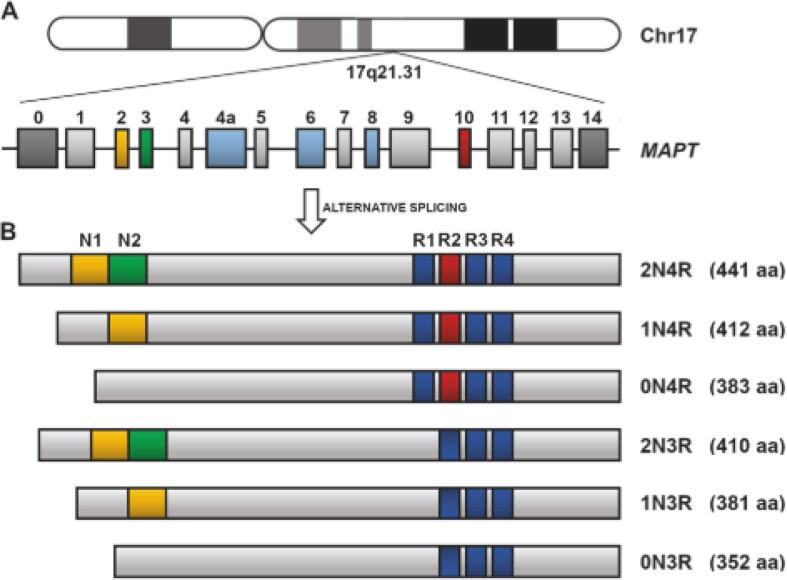
Representation of the human MAPT gene and the tau isoforms produced through alternative splicing. **A.** The MAPT gene is found on chromosome 17 and contains 16 exons, three of which are subjected to alternative splicing. E2, E3, AND E10 undergo alternative splicing which results in six isoforms in the adult human brain. **B.** The six tau isoforms that exist differ in terms of both *N*-terminal inserts and C-terminal repeats, the latter of which confers the designation of 3R or 4R tau. *Figure is reproduced from Didonna A. Tau at the interface between neurodegeneration and neuroinflammation. Genes Immun 21: 288–300, 2020.*

**Fig. 2:**
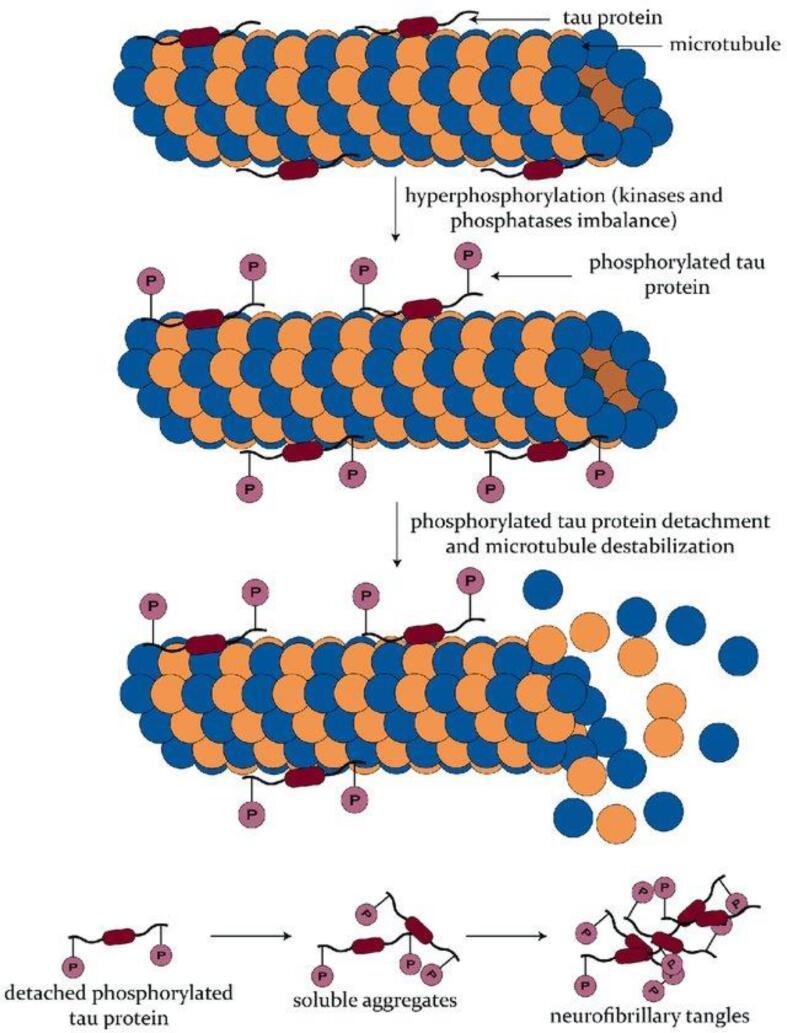
Schematic of the process of neurofibrillary tangle (NFT) formation. Under normal conditions, tau acts as a microtubule-associated protein. Aggregation-prone pathological tau becomes hyper-phosphorylated, ultimately leading to microtubule destabilization through dissociation. Soluble phosphorylated tau proteins come together to form NFTs. *Figure is reproduced from Balasa, Adrian & Chircov, Cristina & Grumezescu, Alexandru. (2020). Body Fluid Biomarkers for Alzheimer’s Disease—An Up-To-Date Overview. Biomedicines. 8. 421. https://doi.org/10.3390/biomedicines8100421*.

**Fig. 3:**
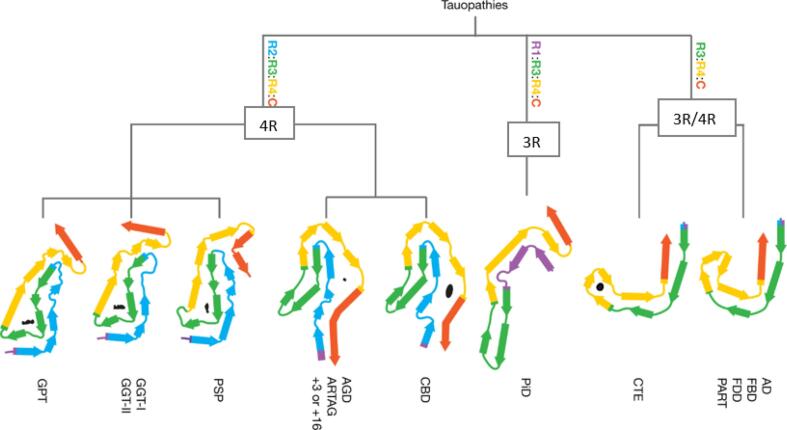
Structural classification of tauopathies based on cryogenic electron microscopy (cryo-EM). Tau residues are depicted by colouration and listed from R1-R4. Tau filament composition is classified according to 1) composition by isoforms and 2) core layer structure. Among the 3R/4R tauopathies, the CTE tau fibril is distinct from AD, FBD, FDD, and PART fibrils. Similarly, the 4R tauopathies are divided into two classes with either three-layered core regions (PSP, GPT, and GGT) or four-layered core regions (CBD, ARTAG, and AGD). AD = Alzheimer’s disease, FBD = familial British dementia, FDD = familial Danish dementia, PART = primary age-related tauopathy, CTE = chronic traumatic encephalopathy, PiD = Pick’s disease, CBD = corticobasal degeneration, AGD = argyrophilic grain disease, ARTAG = ageing-related tau astrogliopathy, PSP = progressive supranuclear palsy, GGT = globular glial tauopathy, GPT = GGT-PSP-tau.  *Figure is modified from Shi Y, Zhang W, Yang Y, Murzin AG, Falcon B, Kotecha A, van Beers M, Tarutani A, Kametani F, Garringer HJ, Vidal R, Hallinan GI, Lashley T, Saito Y, Murayama S, Yoshida M, Tanaka H, Kakita A, Ikeuchi T, Robinson AC, Mann DMA, Kovacs GG, Revesz T, Ghetti B, Hasegawa M, Goedert M, Scheres SHW. Structure-based classification of tauopathies. Nature. 2021 Oct;598(7880):359*–*363. https://doi.org/10.1038/s41586-021-03911-7. Epub 2021 Sep 29. PMID: 34588692; PMCID: PMC7611841.*

Tau is a microtubule-associated protein (MAP) that is predominantly found in the axon of neurons [13]. It normally functions to facilitate protein movement through aiding in microtubule stabilization [14]. There are various isoforms of tau produced through alternative splicing: some have 3 tandem repeat sequences (3R tau), and some have 4 (4R tau) (Fig. 1) [15]. The ratio of 3R-to-4R tau is approximately equal in healthy individuals but becomes disproportionate under pathological conditions [16]. Tau pathology occurs due to hyperphosphorylation. Normally, phosphorylation of tau permits its localization to neuronal soma but hyperphosphorylation disrupts microtubule functionality and ultimately leads to the formation of neurofibrillary tangles (NFTs) in neurons and glial cells (Fig. 2) [17]. NFTs are aggregates of hyperphosphorylated tau that comprise different isoforms and take on various conformations, depending on the tauopathy (Fig. 3).

### 2.1 Tau and parkinsonisms

The PSP- and CBD-tau aggregates predominantly consist of 4R tau which has 4 tandem repeat sequences of 31 or 32 amino acids at the C-terminus [17]. With parkinsonisms, pathological tau deposits are found both intracellularly in neurons and in astrocytes and oligodendrocytes [18], [19]. The structural conformation of 4R tau NFTs differ slightly between CBD and PSP, with 4- and 3-layered folds at the core respectively (Fig. 3) [20]. Furthermore, the differential distribution of tau pathology is evident: pathological tau is localized to the midbrain in PSP versus the cortical pathology observed in CBD [21], [22].

PSP neuropathology is hallmarked by tufted astrocytes and straight and tubular NFTs [9]. The brain regions with the highest tau burden, and therefore posited to be those initially affected by PSP pathology, are the globus pallidus, subthalamic nucleus, and substantia nigra [9]. Although to a lesser degree, pathological tau is also found in cortical areas and the dentate nucleus [23].

The neuropathology of autopsy-confirmed CBD cases includes astrocytic plaques and degeneration in the cerebral white matter, substantia nigra, and globus pallidus contralateral to the clinically more affected side. Additionally, these confirmed cases of CBD also present with argyrophilic threads in cerebral white matter and cortex and “ballooned neurons” [10], [24].

## 3 Imaging pathological tau

Over the past decade, advances in neuroimaging techniques and the development of various radiotracers have allowed researchers to image tau in tauopathies. This work is most abundant in the realm of AD, but there is growing literature for APs. The differentiation between APs and PD is crucial as they have different prognoses and therefore respond differentially to therapeutic treatments. Particularly, patients with APs typically demonstrate levodopa resistance with little benefit from the drug [25]. Because of this, there are a few therapeutic strategies being developed with the aim of targeting pathological tau through immunotherapy [8]. Therefore, it is important to identify a first-in-class tau PET tracer that is best able to localize AP-tau pathology for accurate targeting in these clinical trials.

PET is a non-invasive imaging technique that uses radioligands to bind to various targets in vivo. The radioligands are typically labelled with radioisotopes such as carbon-11 or fluorine-18 which have relatively short half-lives, making them optimal for clinical and experimental imaging. As with other brain-targeted drugs, tau PET radiotracers need to be lipophilic to cross the blood–brain-barrier and penetrate the plasma cell membrane since tau is an intracellular protein [26]. Additionally, those developed thus far have had low molecular weights coupled with rapid uptake and washout from the brain to limit off-target binding [27].

### 3.1 First generation tracers

#### 3.1.1 [18F]AV-1451 (flortaucipir)

[18F]AV-1451 (or flortaucipir) was among the first of the tau PET tracers developed and consequently is the most widely studied. Amid preliminary studies using this tracer, researchers found that it demonstrates highly selective binding to tau over amyloid-beta [28]. More recently, there has been a shift in research using this tracer and a summary of recent [18F]AV-1451 studies can be found in Table 1.

**Table 1:** Main findings from [18F]AV-1451 tau PET studies.

Paper	Tracer	Clinical population	Findings
Goodheart (2021)	[18F]AV-1451	11 CBS, 2 CBD	Asymmetric tracer retention in basal ganglia and cortical motor regions, consistent with CBD pathology
Holland (2021)	[18F]AV-1451	23 PSP, 12 CBS, 19 HC	Tracer binding decreased in regions with lower synaptic density; overall low affinity of [18F]AV-1451 for 4R-tau
Ghirelli (2020)	[18F]AV-1451	10 PSP, 10 CBD	Binding patterns displayed efficacy in differentiating autopsy-confirmed CBD and PSP; further work with larger sample sizes needed to verify results
Malpetti (2020)	[18F]AV-1451	17 PSP-RS, 15 HC	Increased binding in tau-specific regions; highest binding in pons, medulla, and basal ganglia
Nicastro (2020)	[18F]AV-1451	23 probable PSP, 23 HC	Increased tracer binding in tau-specific regions; tracer binding correlated with grey matter loss in frontal regions; off-target binding observed in neuromelanin-containing cells in substantia nigra and MAO in striatum
Soleimani-Meigooni (2020)	[18F]AV-1451	8 AD, 10 non-AD tauopathies (4 PSP, 3 CBD), 3 non-tau FTLD	Tracer binding in accordance with Braak tau pathology in AD; unable to accurately differentiate non-AD tauopathies
Tsai (2019)	[18F]AV-1451	11 nfvPPA, 10 CBS, 10 bvFTD, 2 svPPA, 12 MAPT mutation carriers	Elevated binding in frontal white matter; limited sensitivity for non-AD tau
Whitwell (2019)	[18F]AV-1451	105 PSP, 30 HC	Increased uptake in striatum, globus pallidus, and thalamus; different PSP variants showed different retention patterns
Whitwell (2019)	[18F]AV-1451	16 PSP, 39 HC	Tracer binding less effective than changes in midbrain atrophy as a longitudinal biomarker in PSP
Ali (2018)	[18F]AV-1451	14 CBS	Significant tracer uptake was only seen in beta-amyloid-positive patients; [18F]AV-1451 may have little specificity for non-AD tauopathies
Coakeley (2018)	[18F]AV-1451	1 CBD, 10 HC	[18F]AV-1451 preferentially binds to PHF tau over straight filaments in CBD
Niccolini (2018)	[18F]AV-1451	11 CBS, 20 HC, 33 MCI due to AD	Asymmetric tracer binding in CBS patients, consistent with disease pathology
Cho (2017)	[18F]AV-1451	14 PSP, 15 PD, 15 HC	Increased tracer uptake in subcortical regions in PSP, consistent with pathology; tracer binding did not correlate with clinical severity in PSP or PD
Cho (2017)	[18F]AV-1451	6 CBS, 20 HC	Asymmetric tracer uptake in putamen, globus pallidus, and thalamus contralateral to clinically more affected side; asymmetric binding also found in motor-related subcortical gray and white matter structures
Coakeley (2017)	[18F]AV-1451	6 PSP, 6 PD, 10 HC	No significant differences in tracer uptake amongst all groups; [18F]AV-1451 may be specific for PHF tau in AD
Coakeley (2017)	[18F]AV-1451	6 PSP, 6 PD, 10 HC	Decreased tracer binding in substantia nigra of parkinsonism patients may reflect depigmentation of this region
Hammes (2017)	[18F]AV-1451	1 PSP	Elevated tracer binding in PSP patient; results inconclusive regarding affinity for AP-tau over AD-tau
Marquié (2017)	[18F]AV-1451	2 PSP, 1 MAPT carrier	Increased tracer uptake in substantia and midbrain did not correlate with autoradiographic signal
Schonhaut (2017)	[18F]AV-1451	33 PSP, 26 PD, 46 HC	Increased tracer binding to PSP-specific subcortical regions; binding in globus pallidus best distinguished PSP from PD and HC; decreased substantia nigra binding in PD compared to controls may indicate loss of neuromelanin-containing cells
Smith (2017)	[18F]AV-1451	1 PSP	Off-target binding observed in basal ganglia and brainstem
Josephs (2016)	[18F]AV-1451	1 CBD	Increased tracer binding in accordance with CBD pathology; autoradiography signal failed to detect 4R tau
Mcmillan (2016)	[18F]AV-1451	1 CBD	Tracer binding in accordance with CBD pathology; binding correlates with disease progression
Ono (2017)	[18F]AV-1451	In vitro	Relatively weak [18F]AV-1451 binding to PSP tau deposits in comparison to [11C]-PBB3

Over the past 5 years, there has been growing literature surrounding the application of [18F]AV-1451 in imaging 4R tauopathies, specifically PSP. Overall, there are mixed results concerning whether [18F]AV-1451 uptake in vivo correlates with the neuropathological distribution of tau characteristic to this disease. A recent study that lends support to this tracer’s efficacy in imaging PSP-tau involves 17 patients diagnosed with probable PSP and 29 controls [29]. The participants underwent both [11C]PK11195 PET and [18F]AV-1451 PET, the former being a radiotracer for neuroinflammation. Overall, the aim of this study was to determine whether tau pathology and neuroinflammation colocalize and correlate with PSP clinical severity. The researchers report that [18F]AV-1451 tracer binding is significantly increased in the putamen, thalamus, midbrain, and dentate nucleus in the PSP population compared to controls. Furthermore, tau pathology and neuroinflammation do indeed colocalize in PSP patients, with changes in tracer binding presenting links to clinical severity.

It has also been found that [18F]AV-1451 uptake can differentiate PSP from CBD. Specifically, heightened [18F]AV-1451 retention is observed in the midbrain and left dentate nucleus of PSP patients compared to controls, CBD patients, and other frontotemporal lobe dementia (FTLD) patients [30]. Additionally, [18F]AV-1451 uptake is increased in the red nucleus and cerebellar dentate when comparing PSP to CBD patients alone. Conversely, [18F]AV-1451 uptake is heightened bilaterally in the premotor and motor cortices of CBD patients compared to controls and globus pallidus compared to both PSP and controls. This bilateral distribution of [18F]AV-1451 represents the asymmetrical presentation of CBD symptomology and neuropathology [10], [22].

Although there are several studies lending support to the use of [18F]AV-1451 for imaging tau in 4R tauopathies [10], [22], [29], [30], [31], [32], [33], [34], [35], [36], [37], [38], some others suggest that [18F]AV-1451 may not be the best fit for these circumstances [39], [40], [41], [42], [43], [44], [45], [46], [47], [48], [49], [50], [51]. The main issue found with the use of this tracer in imaging 4R tau has been the lack of specificity and mild response to this conformation in comparison to the mixed 3R/4R paired helical filament (PHF) tau found in AD. Through characterizing the binding profiles of various tau PET tracers, researchers concluded that [18F]AV-1451 has a higher affinity towards 3R and mixed 3R/4R tau fibrils [47], [52]. Specifically, this radiotracer binds to AD-tau fibrils with high affinity and shows little and inconsistent binding to 4R AP-tau fibrils. Additionally, it is demonstrated that [18F]AV-1451 displays one of the highest values for affinity to monoamine oxidase (MAO)-B, indicating that the issue of off-target binding is pronounced with this tracer [47], [52].

#### 3.1.2 [18F]FDDNP, [18F]THK derivatives, and [11C]PBB3

As with most first-generation tau PET tracers, [18F]FDDNP was first developed to image the PHF and amyloid-beta plaques found in AD patients. Recent work conducted with this tracer is summarized in Table 2. Despite its original use, an early study found that there is increased [18F]FDDNP binding in the midbrain, subthalamic regions, and cerebellar white matter of PSP patients [53]. Furthermore, subcortical and cortical tracer uptake also increases with disease severity [53]. Altogether these results lend support to the use of [18F]FDDNP PET as a tool for imaging changes in PSP-tau pathology, although researchers caution this use due to the tracer’s lack of specificity.

**Table 2:** Main findings from other first-generation tau PET studies.

Paper	Tracer	Clinical population	Findings
Schröter (2020)	[11C]PBB3	2 CBS, 7 PSP, 2 AD	Binding consistent with tau pathology in CBS, PSP, and AD
Ono (2017)	[11C]PBB3	In vitro	Increased tracer binding to PSP tau sites
Chiotis (2018)	[18F]THK-5317	16 AD, 2 CBS	Tracer retention increased in CBS patients with disease progression, especially in basal ganglia and frontotemporal areas
Chiotis (2016)	[18F]THK-5317	1 CBD, 1 PSP, 9 AD, 13 MCI, 5 young HC, 4 old HC	Tracer binding consistent with CBD and PSP pathology; different distribution from AD patients
Ng (2017)	[18F]THK-5351	5 MCI, 2 AD, 1 PSP	Tracer uptake is reduced by MAO-B inhibitor, especially in the basal ganglia and thalamus
Kepe (2013)	[18F]FDDNP	15 PSP, 9 PD, 5 HC	Binding consistent with PSP pathology; increased subcortical and cortical involvement with disease progression

These original concerns are echoed by a more recent study in which researchers examined PD patients with and without dementia to determine if increased tau burden correlates with cerebrospinal fluid (CSF) amyloid-beta and tau [54]. Overall, there is higher [18F]FDDNP binding in lateral temporal regions for affected patients with dementia compared to those without [54]. Although researchers sought out to image tau using this tracer, they believe that this increased signal is due to amyloid-beta over tau because of the concurrent decrease in CSF amyloid-beta observed. As evidenced by these two studies, the main concern of imaging AP-tau using [18F]FDDNP is its affinity for amyloid-beta.

[18F]THK-5351, [18F]THK-5317, and [18F]THK-5117 are the arylquinoline derivatives. Each of these tracers have their own benefits and downfalls, which are summarized in Table 2. Early work with these tracers was predominantly conducted using [18F]THK-5317 and [18F]THK-5117. Both tracers showed promising results for imaging AP-tau in vitro [48] and in vivo [55], [56]. Overall the findings point to the greater clinical utility of [18F]THK-5317 because it is able to distinguish CBD and PSP patients from AD patients, albeit with a lesser sensitivity than [18F]AV-1451 [55], [56].

Despite the initial promising findings with the original arylquinoline derivatives, these tracers exhibit significant off-target white matter binding. Consequently, [18F]THK-5351 was developed to address this concern [57], [58]. Using in vivo kinetics and distribution volume ratio (DVR) estimates, researchers found that [18F]THK-5351 displays quicker gray matter and cortical white matter clearance, along with higher estimates in AD-tau regions of interest (ROI) than [18F]THK-5317 [59]. Furthermore, [18F]THK-5351 shows better specificity to PSP-tau than other tau PET tracers [60], [61]: in fact, its uptake was shown to correlate with PSP neuropathology and progressive disease severity [62], [63].

Although initial analyses proposed [18F]THK-5351 as an improved tau PET tracer, this tracer has a high affinity for MAO-B both in vivo and in vitro [58], [64]. Using an MAO-B inhibitor, called selegiline, Ng et al [64] report that [18F]THK-5351 tracer uptake is significantly reduced with the administration of selegiline. In summary, these arylquinoline derivatives do show significant binding to both CBD- and PSP-tau, but the issue of off-target binding raises great concern for their clinical utility.

[11C]PBB3 was the first of the original tau PET tracers to show evidence for binding to a broad spectrum of tau aggregates [65]. It was originally developed to screen for beta-sheet binding capability because of the beta-sheet secondary structure that forms within tau filaments [66]. A summary of recent findings using [11C]PBB3 can be found in Table 2.

When tested in CBS patients, [11C]PBB3 binding is observed extensively in the dorsal frontal, motor, and premotor cortices contralateral to the clinically more affected side [67]. This is consistent with CBD/S pathology which is hallmarked by asymmetric symptomology. In PSP patients, [11C]PBB3 uptake is heightened in the midbrain and thalamus compared to AD and CBS patients [67]. This finding is consistent with the neuropathological distribution of PSP-tau inclusions [9], [68].

Overall, [11C]PBB3 seems like a promising tracer for visualizing the 4R-tauopathies CBD and PSP. However, one study discovered that tracer binding is lower in CBS and PSP patients when compared to AD patients [67]. The researchers propose two explanations for this: (1) tau pathology load is lower in these tauopathies and (2) [11C]PBB3 may have a lower affinity to tufted astrocytes and astrocytic plaques found in PSP and CBD, respectively, than to AD-tau.

### 3.2 Second-generation tracers

#### 3.2.1 [18F]PM-PBB3 ([18F]-APN-1607)

[18F]PM-PBB3 (or [18F]-APN-1607) was recently developed to overcome limitations of [11C] labelled PBB3 such as rapid in vivo metabolism and short half-life [69]. An overview of tau PET studies using [18F]PM-PBB3 can be found in Table 3.

**Table 3:** Main findings from [18F]PM-PBB3 and [18F]PI-2620 tau PET studies.

Paper	Tracer	Clinical population	Findings
Ishizuchi (2021)	[18F]PM-PBB3	1 PSP	Increased tracer retention in PSP tau sites
Li (2021)	[18F]PM-PBB3	20 PSP, 7 MSA, 10 PD, 13 HC	Increased tracer binding in typical PSP regions; binding did not increase with age in any group, indicating unlikely off-target activity
Mashima (2021)	[18F]PM-PBB3	1 CBD	Increased tracer accumulation in subcortical regions and asymmetrical neocortex led to CBD diagnosis
Zhou (2021)	[18F]PM-PBB3	7 MAPT carriers, 15 HC	Tracer binding increased over time as FTD developed in MAPT carriers
Jantarato (2021)	[18F]PI-2620	26 HC, 7 AD, 36 MCI	Tracer binding consistent with Braak staging in AD
Palleis (2021)	[18F]PI-2620	45 CBS, 14 HC	Tracer binding contralateral to clinically more affected side in CBS
Song (2021)	[18F]PI-2620	37 PSP, 10 HC	Globus pallidus internus uptake differentiated PSP from controls
Song (2021)	[18F]PI-2620	10 AD, 15 PSP, 14 CBS	Tracer uptake differentiates 3R/4R tauopathies from PSP and CBD; binding less stable in 4R tauopathies
Tezuka (2021)	[18F]PI-2620	3 PSP, 2 CBS, 1CBD, 8 AD, 7 HC	Increased tracer binding in globus pallidus in APs compared to AD but not HC; late-acquisition PET may not be appropriate for imaging 4R-tauopathies
Brendel (2020)	[18F]PI-2620	40 PSP-RS, 20 PSP-non-RS, 10 alpha-synucleinopathies, 10 AD, 10 HC	Increased binding in PSP target regions compared to control groups; region with best discriminatory ability was globus pallidus internus
Oh (2020)	[18F]PI-2620	3 HC, 9 MCI-AD, 6 FTD, 3 CBS/CBD, 2 PD (with dementia), 3 PSP	Tracer binding distinguished Parkinsonism from other tauopathies; asymmetrical binding in CBD consistent with literature; new off-target binding regions identified

The first in vivo studies using [18F]PM-PBB3 were conducted in FTLD, AD, and control groups [70], [71]. Compared to [18F]AV-1451, [18F]PM-PBB3 effectively images both 3R/4R and 4R tau with increased binding positively correlating with cognitive impairment scores [70]. Furthermore, [18F]PM-PBB3 facilitates accurate diagnosis of FTLD with P301L mutation, lending support to the use of this tracer in reliable in vivo detection of 4R tau. Overall, [18F]PM-PBB3 shows no off-target binding in the basal ganglia and thalamus or with MAO-A and -B [71]. These findings propose that [18F]PM-PBB3 is a promising tracer for imaging AD and potentially other non-AD tauopathies like PSP and CBD.

**Fig. 4:**
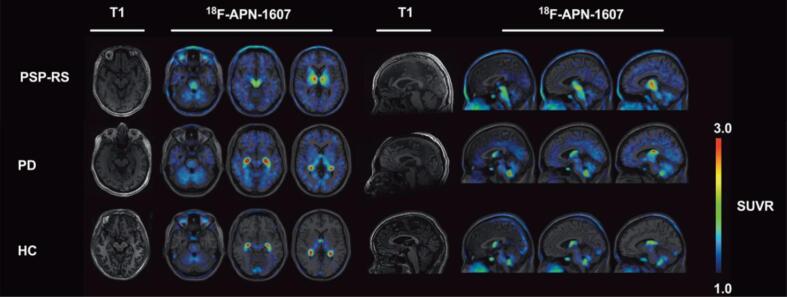
Representative [18F]-APN-1607 (or [18F]PM-PBB3) PET images superimposed on T1 MRI in PSP patients versus PD and controls. Compared to PD and HC, patients with PSP display heightened tracer binding in the midbrain, basal ganglia, subcortical nuclei, and brainstem. The colour scale represents standardized uptake value ratio (SUVR) with cerebellar cortex as the reference regions. PSP-RS = progressive supranuclear palsy-Richardson’s syndrome, PD = Parkinson’s disease, HC = healthy controls. *Figure modified from Li L, Liu FT, Li M, Lu JY, Sun YM, Liang X, Bao W, Chen QS, Li XY, Zhou XY, Guan Y, Wu JJ, Yen TC, Jang MK, Luo JF, Wang J, Zuo C; Progressive Supranuclear Palsy Neuroimage Initiative (PSPNI). Clinical Utility of 18F-APN-1607 Tau PET Imaging in Patients with Progressive Supranuclear Palsy. Mov Disord. 2021 Oct;36(10):2314*–*2323. https://doi.org/10.1002/mds.28672*. *Epub 2021 Jun 5. PMID: 34089275.*

Additional evidence for [18F]PM-PBB3′s utility in imaging 4R tau has been uncovered over the past year [72], [73], [74], [75]. One study reports that increased tau accumulation over time corresponds with significant [18F]PM-PBB3 uptake in patients with MAPT mutations [72]. Overall, the researchers conclude that [18F]PM-PBB3 is more effective than first-generation tracers in binding to 4R tau and tracking the accumulation of this isoform over time in MAPT mutation carriers. Lending similar support, the first antemortem study using [18F]PM-PBB3 in PSP patients displays that tracer retention is heightened in the midbrain, subthalamic nucleus, and cerebellar dentate nucleus of these patients [73]. In addition to these regions, Li et al [74] report increased tracer binding in the striatum, putamen, globus pallidus, thalamus, tegmentum, substantia nigra, pontine base, red nucleus, raphe nuclei, and locus coeruleus (Fig. 4). Furthermore, clinical severity of PSP is indicated by the magnitude of tracer binding in the subthalamic nucleus, midbrain, substantia nigra, red nucleus, pontine base, and raphe nucleus [74]. Similar success was observed in the only study using this tracer in CBD: researchers find that [18F]PM-PBB3 accumulation is pronounced in the occipital cortex, basal ganglia, thalamus, midbrain, subcortical region, and asymmetrical neocortex of CBD patients [75].

As with most tau PET tracers tested in 4R-tauopathies, it is important to consider off-target binding. In the studies conducted thus far, [18F]PM-PBB3 uptake does not correlate with age in all groups assessed, which suggests that off-target activity due to normal aging is not likely [74], though this should be investigated further. Taken together, these results point towards the great clinical utility of [18F]PM-PBB3 in early diagnosis of non-AD tauopathies. Although these outcomes are promising, further work with greater sample sizes, especially in CBD patients, is needed to validate these findings.

#### 3.2.2 [18F]PI-2620

[18F]PI-2620 was originally discovered when Kroth et al [76] identified a new core structure for tau PET tracers called pyrrolo[2,3-*b*:4,5-*c*’]dipyridine. Out of all compounds surveyed, [18F]PI-2620 showed the most favorable results due to its quick washout and affinity for tau aggregates reflective of Braak staging tau pathology progression [76]. Additionally, [18F]PI-2620 does not demonstrate off-target binding to MAO-A or -B, substantia nigra, basal ganglia, or choroid plexus, unlike a few other potential tracers [59], [77]. An overview of studies using [18F]PI-2620 PET can be found in Table 3.

In these studies, researchers report that the best image quality and signal-to-noise ratio of this tracer is produced at 45–75 min post-injection in AD patients [78], [79]. Furthermore, non-invasive static [18F]PI-2620 PET scans during this period provides comparable quantification accuracy to dynamic scans with arterial sampling in this cohort [74]. Although these are the optimal conditions for [18F]PI-2620 PET in AD, recent work has found that [18F]PI-2620 tracer kinetics differ in 4R-tauopathies, presenting with earlier peaks than in AD [80].

**Fig. 5:**
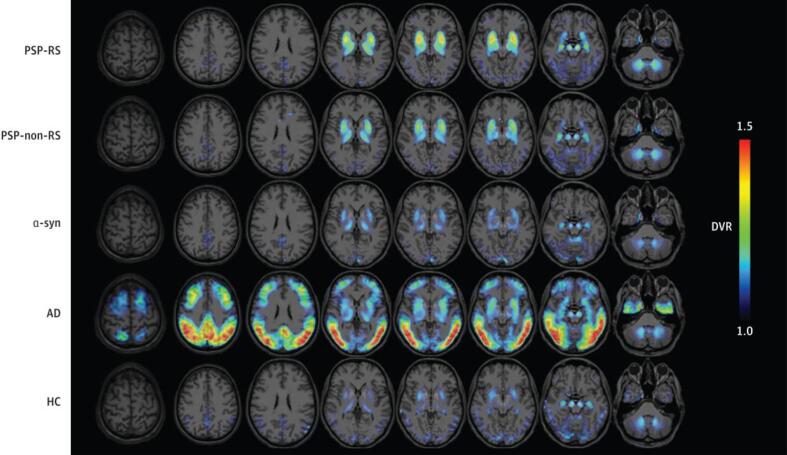
Representative [18F]PI-2620 distribution volume ratio (DVR) binding maps superimposed on standard MRI imaging templates for all disease cohorts. Differential tracer binding effectively distinguishes study groups. Binding is highest in the globus pallidus for PSP groups. PSP-RS = progressive supranuclear palsy-Richardson’s syndrome, PSP-non-RS = progressive supranuclear palsy-non-Richardson’s syndrome, a-syn = alpha-synucleinopathies, AD = Alzheimer’s disease, HC = healthy controls. *Figure is reproduced from Brendel M, Barthel H, Van Eimeren T, Marek K, Beyer L, Song M, Palleis C, Gehmeyr M, Fietzek U, Respondek G, Sauerbeck J, Nitschmann A, Zach C, Hammes J, Barbe MT, Onur O, Jessen F, Saur D, Schroeter ML, Rumpf JJ, Rullmann M, Schildan A, Patt M, Neumaier B, Barret O, Madonia J, Russell DS, Stephens A, Roeber S, Herms J, Bötzel K, Classen J, Bartenstein P, Villemagne V, Levin J, Höglinger GU, Drzezga A, Seibyl J, Sabri O. Assessment of 18F-PI-2620 as a Biomarker in Progressive Supranuclear Palsy. JAMA Neurol 77: 1408–1419, 2020.*

Although this tracer’s concentration and stability is lower in 4R tauopathies, [18F]PI-2620 does appear to be a promising tracer for distinguishing parkinsonisms with improved accuracy. For instance, [18F]PI-2620 uptake is asymmetric in the pallidum of CBD patients compared to PD and PSP patients [81]. Furthermore, this tracer has the potential for great clinical utility in monitoring CBS disease progression because the hemispheric lateralization of [18F]PI-2620 is associated with the contralateral manifestation of disease severity [82]. In terms of PSP, Brendel et al [83] demonstrate [18F]PI-2620′s utility in facilitating more reliable diagnoses of this disorder. In their study, they use DVR measurements and autoradiography to show that this tracer is effective in differentiating PSP from alpha-synucleinopathies (e.g., PD), AD, and controls with 85 % sensitivity and 77 % specificity (Fig. 5). Consistent with previous literature, the region with the most significant difference is the globus pallidus internus in PSP patients compared to controls.

Although [18F]PI-2620 is shown to be an effective tracer in imaging 3R/4R tau in AD and distinguishing these patients from mild cognitive impairment (MCI) patients and controls [84], more work is needed to lend similar support to its utility in APs. Thus far, studies using this tracer demonstrate improvements in off-target binding to the basal ganglia, white matter, and cortex [81], [85], but new areas of non-specific binding have been identified. For example, Oh et al [81] discovered non-specific binding in the choroid plexus, cerebral venous sinus, and meningioma. At this point, the in vivo [18F]PI-2620 studies are limited and further research is required to draw conclusions regarding its efficacy in imaging AP-tau pathology.

#### 3.2.3 [18F]RO-948 (RO6958948) and [18F]JNJ-067 ([18F]-JNJ-64326067-AAA)

[18F]RO-948 (or RO6958948) was created with the goal of improving tracking abilities for longitudinal tau distribution in AD patients [86]. [18F]RO-948 presents with superior binding characteristics and kinetic properties in vivo compared to other candidate tracers [86], [87]. In the few studies conducted thus far, tracer performance has mainly been successful in AD patients [88], [89], [90]. A summary of these experiments can be found in Table 4.

**Table 4:** Main findings from other second-generation tau PET studies.

Paper	Tracer	Clinical population	Findings
Ossenkoppele (2021)	[18F]RO-948	673 HC, 443 MCI, 315 CE	[18F]AV-1451 and [18F]RO-948 were able to detect AD-tau, even in preclinical and prodromal stages of AD
Smith (2020)	[18F]RO-948	18 AD, 3 amyloid-beta-positive amnestic MCI, 4 HC	Tracer retention higher in entorhinal cortex but lower in basal ganglia, thalamus, and choroid plexus; SUVR plateaus by the end of scanning interval, indicating less concern for time-dependent biases
Leuzy (2020)	[18F]RO-948	100 CE, 26 PD/PD (with dementia), 6 MSA, 16 PSP, 25 DLB, 7 svPPA, 12 bvFTD, 257 HC, 154 MCI	Elevated tracer binding mostly observed in beta-amyloid-positive cases; provides evidence for [18F]RO-948 specificity for AD-tau
Honer (2018)	[18F]RO-948	In vitro	Tracer binding consistent with Braak staging in AD
Baker (2021)	[18F]JNJ-067	4 HC, 5 MCI, 5 AD, 3 PSP	Significant tracer binding in AD-specific regions only; unlikely that this tracer will be useful in AP research
Gogola (2021)	[18F]MK-6240	5 probable AD, 1 MCI-amnestic, 9 no subjective cognitive complaint	Tracer binding in medial temporal lobe and neocortex consistent with AD pathology; off-target binding observed to similar degrees as [18F]AV-1451
Salinas (2019)	[18F]MK-6240	12 AD, 3 HC	Rapid tracer uptake in AD patients compared to HC; no evidence of off-target binding; high test–retest reliability
Hostetler (2016)	[18F]MK-6240	In vitro	Binding pattern consistent with phosphorylated tau distribution in AD brain slices; favourable tracer kinetics demonstrated
Lindberg (2021)	[18F]CBD-2115	In vitro	High tracer binding affinity in 4R-tau in transgenic mice; human tissue homogenates also showed high tracer specificity for 4R-tau; tracer clearance was rapid, which may serve as an issue with uptake in the future

Leuzy et al [89] present results supporting the use of this tracer in discriminating AD from non-AD tauopathies, including PSP. Overall, they report that [18F]RO-948 tracer retention is highest in amyloid-beta-positive cases, indicating that this tracer may not have a specificity for 4R AP-tau. Although this tracer has shown great promise in AD, one thing to consider in future studies is the off-target binding reported in the skull and meninges [88].

[18F]JNJ-067 (or [18F]-JNJ-64326067-AAA) was developed to improve the accuracy of tau PET imaging by addressing concerns with available tracers. These include inability to reach steady state during PET experimentation which affects the signal-to-noise ratio, off-target binding, and limited affinity for 4R tau [91]. A summary of findings from Baker et al [91] can be found in Table 4. Using PET imaging in controls, amyloid-positive MCI, AD, and PSP patients, these researchers evaluated [18F]JNJ-067′s efficacy in addressing these concerns. Overall, they find that the tracer does not reach steady state in 0–90 min post-injection and there is significant off-target binding in the putamen, pallidum, thalamus, midbrain, superior cerebellar gray, and white matter. Furthermore, [18F]JNJ-067 retention is only observed in AD patients where mini-mental state examination (MMSE) scores correlate with increased binding in the entorhinal cortex and temporal meta-ROI [91]. The lack of binding in other participants indicates that this tracer is unable to pick up on low levels of AD-tau in MCI patients and 4R tau in the PSP group. Therefore, the use of [18F]JNJ-067 for future work in APs is unlikely due to its lack of sensitivity for this cohort demonstrated in preliminary work.

#### 3.2.4 [18F]MK-6240 and [18F]CBD-2115

[18F]MK-6240 and [18F]CBD-2115 are two tracers that have not yet been assessed in patients with parkinsonisms, but a summary of recent relevant studies can be found in Table 4. Preclinical characterization of [18F]MK-6240 was promising due to its in vitro binding pattern: it showed preferential binding to NFT-rich brain homogenates compared to amyloid-rich homogenates [92]. Furthermore, through direct comparison to [18F]AV-1451, researchers report that this tracer displays 5-fold higher sensitivity to NFTs.

In the few in vivo studies conducted, researchers have shown that [18F]MK-6240 exhibits a wide dynamic range of uptake and binding patterns in MCI and AD patients in line with NFT deposition patterns. In accordance with preliminary in vitro findings, most studies conducted have found little to no off-target binding with this tracer [93], [94], although Gogola et al [95] state that [18F]MK-6240 displays off-target binding similar to [18F]AV-1451 in the striatum. New off-target regions of interest are also illuminated in this study, including the retina, ethmoid sinus, and dura mater. The incongruity between in vitro and in vivo findings in this regard demonstrates that the research conducted with this tracer is still in its infancy and further studies with a wider range of tauopathies are necessary to make conclusions about its efficacy. An example of one such study was conducted by Levy and colleagues [96], in which they conducted [18F]MK-6240 PET on patients with FTLDs. Overall, they find that patients with amyloid-negative P301L and R406W MAPT mutations display significant tracer binding to NFTs with limited off-target binding. The binding reported here is quite mild, but the results are significant and highlight the potential for [18F]MK-6240′s utility in imaging non-AD tauopathies.

[18F]CBD-2115 has been assessed as a potential radiotracer for imaging 4R-tauopathies specifically [97]. In human tissue, [18F]CBD-2115 shows similar affinity for AD- and PSP-tau. Conversely this tracer shows high affinity for 4R-tau in P301L transgenic mice brain tissue [97]. This work is still preliminary but initial PET imaging in animal models of PSP show promising results for [18F]CBD-2115 uptake in 4R tau with rapid clearance following experimentation. Future work should focus on improving these initial values and increasing the binding affinity to 4R tau over AD-tau in human tissue and subsequently in vivo.

## 4 Challenges for PET imaging

Thus far, the main challenge with tau PET imaging has been off-target binding and ante- vs post-mortem inconsistencies. Tau aggregates predominantly form intracellularly, which makes them difficult to target using PET [98]. Additionally, tau PET tracers need to be able to differentiate between amyloid beta plaques and NFTs, which is difficult due to their similar beta-sheet conformations. Although the second-generation tracers like [18F]PM-PBB3 and [18F]PI-2620 show improvements with respect to off-target binding, the research supporting their specificity for 4R tau is still in its infancy. Therefore, it is important to continue working with clinical populations to determine if any available radiotracers are effective in imaging non-AD tauopathies and monitoring disease progression. Researchers are searching for an ideal tracer that binds to 4R tau with a high dynamic range, has little off-target binding, achieves steady-state within a half-life, and has low test–retest variability [91]. There are a handful of tau PET tracers that have yet to be tested in APs but show promising results in AD and other related tauopathies. The results of these studies can be found in Table 4. Future work assessing these tracers in vivo in AP patients will be necessary to determine their clinical utility for these diseases.

Another area of investigation in tau PET imaging is the appropriate acquisition time and measurement parameters for these tracers. As evidenced by the work with [18F]PI-2620, the optimal time windows in AD versus non-AD tauopathies may differ [80], [85], [99]. Overall, standardized uptake value ratio (SUVR) and DVR are the most common parameters for describing the binding patterns of these tau PET tracers in the brain. One recent study described a new protocol called the dual-time-window (DTW) in which dynamic scans are separated by 60-minute breaks [100]. Here, the researchers demonstrate that DVR values reported from this protocol prove to be promising with the second-generation tracer [18F]MK-6240. Although this novel tracer has not yet been tested in patients with parkinsonisms, these results support a promising new avenue of assessment that serves to increase patient comfort and retain quantitative accuracy.

## 5 Conclusion

Overall it seems that the second-generation tau PET tracers have overcome off-target binding to the basal ganglia, cerebral white matter, and MAO-A and B. The concern for their specificity to 4R AP-tau over 3R/4R AD-tau is still up for debate, with various conflicting results. Although some of the currently available tau PET tracers lack comparable affinity to AP- and AD- tau, they are still able to distinguish patients with APs from those with other neurodegenerative diseases to some degree. While [18F]PM-PBB3 and [18F]PI-2620 show high efficacy in distinguishing 4R tauopathies, their use introduces new off-target binding regions including the skull and meninges. The literature presented in this review demonstrates the great strides taken in the field of tau PET imaging of APs. Although there have been great improvements from the original studies with first-generation tracers, more research is needed with larger sample sizes to arrive at a conclusion regarding the diagnostic utility of tau PET imaging. Future work should focus efforts on replicating and expanding on the promising findings found with the second-generation tracers. The current diagnostic process for APs is extensive and relies on clinical examination and post-mortem confirmation. Therefore, it is pertinent that this remains an active area of research to establish a reliable ante-mortem diagnostic imaging biomarker that will help clinicians and their patients arrive at a diagnosis earlier on in disease course.

## Declaration of Competing Interest

The authors declare that they have no known competing financial interests or personal relationships that could have appeared to influence the work reported in this paper.
